# PARP inhibitors combined with radiotherapy: are we ready?

**DOI:** 10.3389/fphar.2023.1234973

**Published:** 2023-10-26

**Authors:** Chen Sun, Alan Chu, Rui Song, Shijia Liu, Ting Chai, Xin Wang, Zongwen Liu

**Affiliations:** Department of Radiation Oncology, The Second Affiliated Hospital of Zhengzhou University, Zhengzhou, Henan, China

**Keywords:** PARP inhibitors, radiotherapy, targeted drugs, combination therapy, drug toxicity

## Abstract

PARP was an enzyme found in the nucleus of eukaryotic cells that played a crucial role in repairing damaged DNA. Recently, PARP inhibitors have demonstrated great potential in cancer treatment. Thus, the FDA has approved several small-molecule PARP inhibitors for cancer maintenance therapy. The combination of PARP inhibitors and radiotherapy relies on synthetic lethality, taking advantage of the flaws in DNA repair pathways to target cancer cells specifically. Studies conducted prior to clinical trials have suggested that the combination of PARP inhibitors and radiotherapy can enhance the sensitivity of cancer cells to radiation, intensify DNA damage, and trigger cell death. Combining radiotherapy with PARP inhibitors in clinical trials has enhanced the response rate and progression-free survival of diverse cancer patients. The theoretical foundation of PARP inhibitors combined with radiotherapy is explained in detail in this article, and the latest advances in preclinical and clinical research on these inhibitors for tumor radiotherapy are summarized. The problems in the current field are recognized in our research and potential therapeutic applications for tumors are suggested. Nevertheless, certain obstacles need to be tackled when implementing PARP inhibitors and radiotherapies in clinical settings. Factors to consider when using the combination therapy are the most suitable schedule and amount of medication, identifying advantageous candidates, and the probable adverse effects linked with the combination. The combination of radiotherapy and PARP inhibitors can greatly enhance the effectiveness of cancer treatment.

## 1 Introduction

Radiation therapy is a pivotal modality of oncologic management that uses α-, β-, γ-, and X-rays to engender ionizing radiation (IR), damaging the chemical bonds of deoxyribonucleic acid (DNA) helices via single- and double-strand breaks (Slade, 2020). This culminates in either direct or indirect tumor cell DNA damage, eliciting cancer cell death and, consequently, exerting anti-tumorigenic effects (Huang and Zhou, 2020). However, the efficacy of radiotherapy is compromised by radiation tolerance. The effectiveness of radiation therapy can be enhanced by using radiosensitizers to optimize therapeutic outcomes (Maier et al., 2016). A plethora of studies have highlighted that PARPis could increase the sensitivity of radiation, bolstering the success rate of radiation therapy in the management of lung, esophageal, and pancreatic cancers, among other malignancies (Tuli et al., 2019; Kong et al., 2021; Sheikh et al., 2023).

## 2 PARP family and members

Poly (ADP-ribose) polymerase (PARP), formerly known as ADP-ribosyl transferase (ART), is a key protein implicated in post-translational modification. Its activity is governed by the catalysis of the ADP-ribosylation process (Bock and Chang, 2016). The PARP family encompasses 18 members, divided into four distinct categories according to structural domains. The first category includes DNA-dependent PARPs, namely, PARP1, PARP2, and PARP3, which can be activated by the N-terminal DNA-binding domain in the presence of discontinuous DNA structures. The second category comprises the ring-shaped PARP enzymes, specifically PARP5a and PARP5b (Gibson and Kraus, 2012; Barkauskaite et al., 2015). The PARP protein features a binding platform for protein-protein interactions comprising five anchoring protein repeat sequences. The third category is composed of PARP12, PARP13.1, and PARP13.2, characterized by the presence of a zinc finger domain with a Cys-Cys-Cys-His (CCCH) sequence, which is involved in regulating the binding of PARP to ribonucleic acid (RNA). MacroPARPs, known for their large structural domains, form the fourth category and include PARP9, PARP14, and PARP15. These macroPARPs are recognized for their ability to bind to ADP and its derivatives. The PARP family members, PARP1, PARP2, and PARP5, exert biological effects by catalyzing the poly-ADP-ribosylation of target proteins (Hassa and Hottiger, 2008; Kleine et al., 2008; Sefer et al., 2022). Excluding enzymatically inactive PARP9 and PARP13, the remaining PARP family members can catalyze a single ADP-ribosylation on target proteins, thereby modulating physiological processes (Todorova et al., 2014; Xing et al., 2021). Meanwhile, PARP1 can regulate gene transcription, cell cycle, apoptosis, and inflammatory responses. As a crucial DNA repair enzyme primarily functioning through the base excision repair (BER) pathway, PARP1 represents a promising target for the development of anti-neoplastic agents (Krishnakumar and Kraus, 2010; Li and Yu, 2015).

## 3 Mechanism of anti-tumor action of PARP inhibitors

### 3.1 PARPi and DNA damage repair

DNA damage may arise from sustained exposure of cells to external and internal stimuli. In order to ensure survival, cells have developed intricate and synchronized pathways for DNA damage repair. These pathways can be broadly classified into two categories. The first encompasses nucleotide excision repair (NER), BER, and mismatch repair (MMR), all of which participate in the repair of DNA single-strand breaks (SSBs). The second category includes homologous recombination (HR) and non-homologous end joining (NHEJ), which are instrumental in repairing DNA double-strand breaks (DSBs) (Ciccia and Elledge, 2010; Li and Yu, 2015). The BER pathway is highly dependent on PARP1, which binds to damaged DNA and catalyzes its PARylation and that of H1 histone following SSBs induced by external stimuli or endogenous factors. The PARylation of PARP1 and histones facilitates the recruitment of repair proteins at the DNA damage site and chromatin relaxation, granting spatial access to these proteins. Repairing damaged DNA in the BER process involves proteins such as X-ray repair cross-complementing gene 1 (XRCC1), DNA polymerase, and DNA ligase (Patidar et al., 2020). Among them, XRCC1 serves as a scaffold protein anchoring other repair components. DNA polymerase catalyzes deoxyribonucleotide synthesis at the damaged site to synthesize normal DNA. DNA ligase connects adjacent phosphodiester bonds to bridge the gap between DNA fragments. PARP1 participates in almost the entire process, from detecting SSBs to repairing damages (Ray Chaudhuri and Nussenzweig, 2017).

PARPis effectively inhibit PARP1 activity, leading to unrepaired SSB accumulation and DSB generation (Pommier et al., 2016). Due to the deficiency of BRCA1/2 or other HR-related genes, it is impossible for HR to fix the DNA damage. Tumor cells with BRCA1/2 mutations and HR deficiencies can be selectively targeted by PARPis (Murai et al., 2012). Despite PARPis being primarily recognized for their involvement in DNA repair, they also exhibit additional anti-cancer functions. For instance, they can induce cell cycle arrest, impede the formation of new blood vessels essential for tumor growth, and mediate immune responses (Li et al., 2022).

PARPis have been extensively employed as sensitizers for radiotherapy and chemotherapy drugs since the 1970s, given the notable contribution of PARP1 in DNA single-strand damage repair (Clark et al., 1971). However, subsequent studies demonstrated that although PARPis significantly sensitize chemotherapeutic agents, severe adverse reactions have hindered clinical development (Kummar et al., 2011; Bendell et al., 2015; Middleton et al., 2015; Oza et al., 2015).

### 3.2 Synthetic lethality(SL)

In 2005, the concept of SL provided a vital theoretical foundation for the use of PARPis as monotherapy in patients diagnosed with HR repair-deficient tumors (Bryant et al., 2005; Oza et al., 2015). SL is typically classified into two categories: unconditional/primitive SL and conditional SL. The former is further sub-divided into gene level, functional pathway level, and organelle level, based on the specificity of its biological mechanism (Li et al., 2020).

#### 3.2.1 Unconditional SL

##### 3.2.1.1 Genetic level

Given that BRCA gene products play a decisive role in HR in the DSB repair process, inhibition of additional DNA repair systems is hypothesized to be fatal in the event of BRCA gene function loss. PARPis, shown to participate in BER, were found to be highly synthetically lethal in BRCA1 and BRCA2 gene mutations (Holloman, 2011).

Following extensive clinical research, the PARPi olaparib was approved for the treatment of BRCA-mutated ovarian cancer at the end of 2014. The notion that tumors with functional HR are sensitive to PARPis signals that cells deprived of other enzymes involved in HR may also be hypersensitive to PARPis. Indeed, defects in genes encoding TP53, WEE1, RAD51, RAD54, DSS1, RPA1, NBS1, ATR, ATM, CHK1, CHK2, FANCD2, FANCA, or FANCC have also been found to confer sensitivity to PARP inhibition (Setton et al., 2021).

##### 3.2.1.2 Functional pathway level

The functional pathway level refers to the expression of SL at the molecular and cellular levels, which can be divided into single-pathway SL, dual-pathway SL, and multi-pathway SL (Li et al., 2020).

Single-pathway SL focuses on a single pathway wherein functionally related genes are sequentially translated into proteins, forming pathways that perform essential functions intracellularly. In many cases, several components of such pathways are complexes formed by the coordinated expression of multiple genes (Neiger et al., 2021). Abnormalities in two or more genes coding for the same protein complex in a pathway can lead to cell death (Dariane and Timsit, 2022).

Dual-pathway SL involves two or more genes and two pathways. Specifically, both pathways perform the same survival function to maintain cell viability, and aberrations in two or more genes that act as key regulatory points in both pathways lead to synthetic lethal interactions in tumors. Conversely, genetic anomalies in only one pathway can still sustain cell survival (Wang et al., 2022).

Multi-pathway SL occurs when pathways form networks to maintain cell viability. The presence of abnormal (mutated, overexpressed, or suppressed) genes in each pathway leads to cell death. However, cells can still survive in the presence of abnormal genes in some, but not all, pathways (Lau et al., 2022).

##### 3.2.1.3 Organelle synthetic lethality

Targeting organelles with synthetic lethality is a more macroscopic approach than synthetic lethal interactions within genes or functional pathways (Genini et al., 2017). This type of SL focuses on influencing or exploiting the primary function of organelles to cause tumor cell death. Currently, various experiments on SL are directed at mitochondrial function (Nguyen et al., 2022).

#### 3.2.2 Conditional SL

Conditional SL represents a specialized form of synthetic lethal effect on tumor cells, influenced by both internal and external conditions such as specific genetic backgrounds, hypoxia, high reactive oxygen species levels, and DNA-damaging agents (Nguyen et al., 2022). It may account for the variation in synthetic lethal effects observed in distinct tumor cells or cell lines of the same cancer type. Following resistance to synthetic lethal tumor-targeting drugs, conditional SL could provide insights into potential solutions. In summary, conditional SL is one step further from nonconditional/original SL and holds promising prospects for the treatment of tumors with various complex conditions in the future.

**FIGURE 1:**
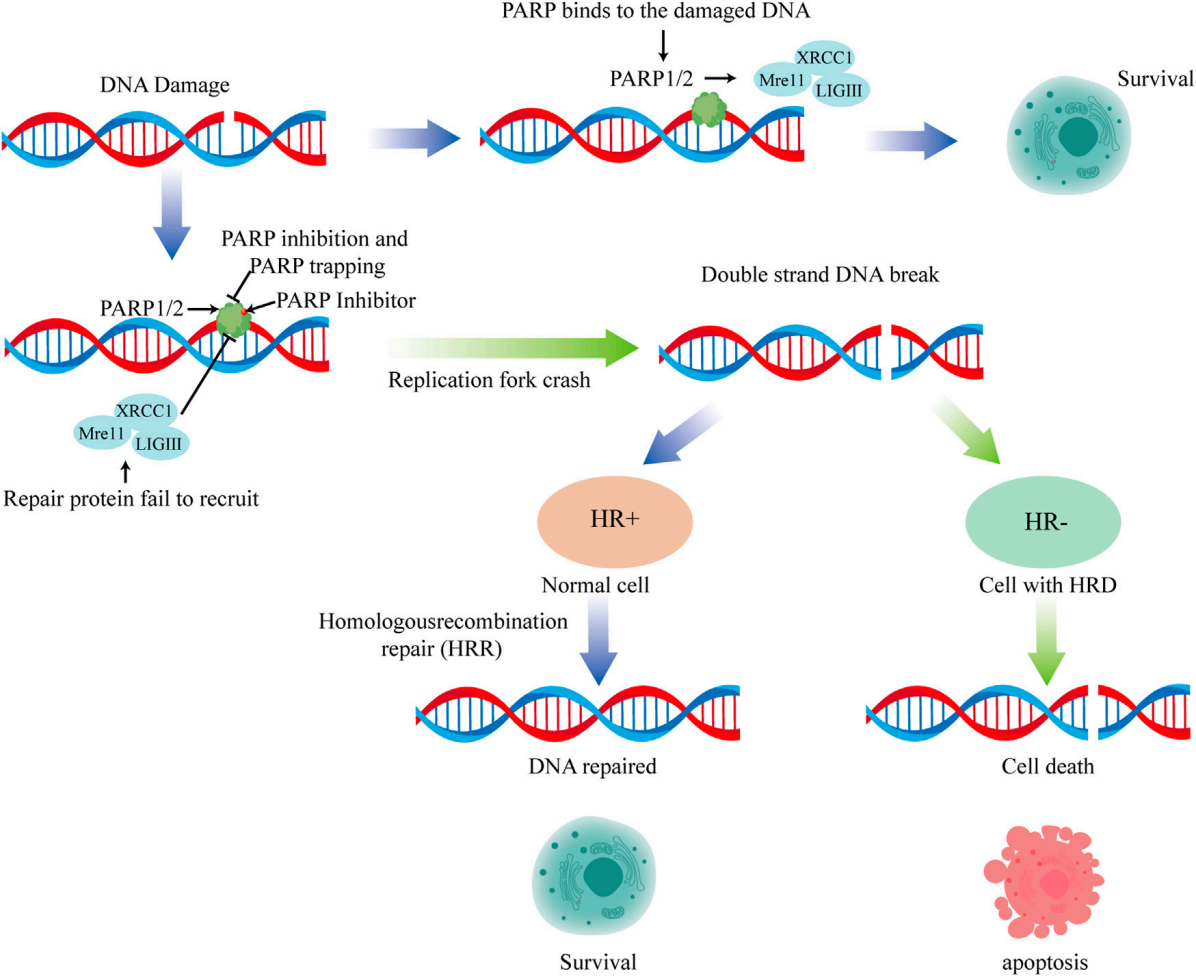
The anti-tumor mechanism of PARP inhibitors.

The SL theory suggests that PARPis can inhibit the repair of DNA single-strand damage by inhibiting the BER pathway, thereby generating replication-dependent DNA double-strand damage when unrepaired DNA single-strand damage encounters a progressing replication fork. In healthy cells, replication-dependent DNA double-strand damage can be repaired via HR. However, cell death due to the inability to timely repair DNA double-strand damage occurs in tumor cells with HR repair defects regulated by mutations in breast cancer susceptibility genes 1/2 (BRCA1/2), among others. In 2012, the PARP1-DNA trapping theory added new dimensions to the mechanism of action of PARPis, suggesting that PARP1 can bind to damaged DNA (Hopkins et al., 2015). PARPis can prevent its detachment from the damaged DNA, thereby forming a stable complex by anchoring to the damaged DNA. This stable complex formation can inhibit other repair-related proteins from binding to and repairing the damaged DNA, resulting in cell toxicity (Figure 1).

## 4 Sensitization effect of PARP inhibitors on radiotherapy

The number of SSBs created by radiation therapy within mammalian cells is 25 times greater than DSBs (Hopkins et al., 2015). Typically, the induced SSBs are rapidly and effectively processed without much impact on cell death. DSBs are the leading cause of cell death induced by radiation therapy. Unrepaired DSBs can cause cell mortality by inducing mitotic failure when irradiated cancer cells attempt to progress via cell division. PARPi delays BER and converts some non-lethal SSBs produced by radiation therapy into lethal DSBs using DNA replication forks, thereby increasing the difficulty of DNA damage repair (Rabenau and Hofstatter, 2016; Derby et al., 2022). Thus, some researchers have studied using PARPi to increase the sensitivity of tumors during radiation therapy.

**FIGURE 2:**
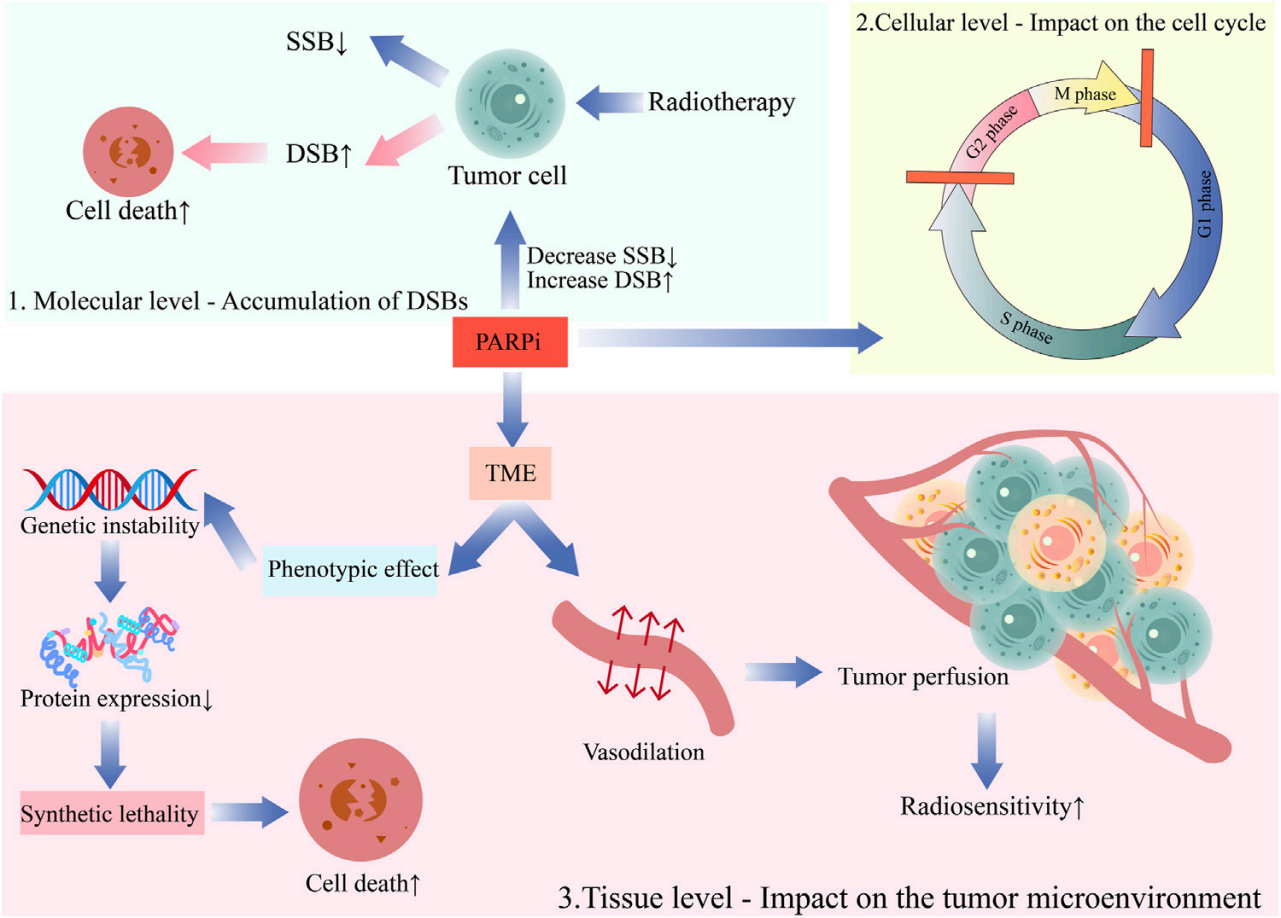
The mechanism of the combination of PARP inhibitors and radiotherapy.1. Molecular level—Accumulation of DSBs; 2. Cellular level—Impact on the cell cycle; 3. Tissue level—Impact on the tumor microenvironment.

The chemotherapy sensitization effect of PARP inhibitors has been preliminarily verified in clinical practice with good efficacy. Additionally, PARP inhibitors have a specific sensitization effect on radiotherapy, with unclear mechanisms. The following are some of the main viewpoints (Figure 2).

### 4.1 Molecular level—accumulation of DSBs

PARPs played a critical role in various DNA repair mechanisms, such as base excision repair (BER), homologous recombination (HR), conventional non-homologous end joining (c-NHEJ), and alternative non-homologous end joining (alt-NHEJ) (Schultz et al., 2003; Mansour et al., 2010; Weigert et al., 2020). DNA is critical for radiobiological effects (for example, cell death, mutation, and carcinogenesis). After SSBs appear in intact DNA, they are easily repaired with the complementary strand on the opposite side as a template. However, DSBs can lead to chromosome breakage into two sections that cannot continue replicating. Therefore, DSBs are the most critical damage due to ionizing radiation on chromosomes (Soni et al., 2018). DNA damage repair is primarily responsible for the radiation resistance of tumor cells. PARP inhibitors can inhibit SSB repair due to radiation, transforming unrepaired SSBs into DSBs in tumor cells. This accumulates DSB damage in DNA that is difficult to repair and promotes tumor cell death. This is the first and foremost radiosensitizing effect of PARP inhibitors (Lesueur et al., 2017).

Bridges et al. conducted a study on lung, breast, and prostate cancer cell lines, which demonstrated that the PARP-1 inhibitor niraparib is both effective and selective in radiosensitizing human tumor cells *in vitro*. The mechanism by which PARP inhibitors (PARPi) radiosensitized cancer cells involved converting sub-lethal single-strand DNA breaks (SSBs) into lethal double-strand DNA breaks (DSBs) during DNA replication, leading to the accumulation of DNA damage and ultimately cell death. This effect was particularly pronounced in cells with defects in homologous recombination repair (HR), due to the concept of “synthetic lethality” (Bridges et al., 2014). Loser et al. described that the radiosensitizing effect of PARPi is more enhanced for rapidly dividing or DNA repair-deficient tumors (Löser et al., 2010). Wurster demonstrated that the radiosensitization due to PARP1 inhibition depends on the HR ability. PARP1 inhibitors impeded DNA replication and improve radiotherapy in HR-deficient HNSCC (Löser et al., 2010).

### 4.2 Cellular level—impact on the cell cycle

The radiosensitivity of cells varies throughout the cell cycle. Cells in or approaching the M-phase are the most radiosensitive. Cells in the G2-phase are generally more sensitive, with similar sensitivity to M-phase cells, while cells in the late S-phase typically demonstrate increased radiation resistance. PARPis can arrest tumor cells in the radiation-sensitive G2/M phase and operate synergistically with radiotherapy (Rae and Mairs, 2017). Additionally, PARPis exert a radiosensitizing effect in the S-phase. Dungey et al. studied the radiation sensitivity of glioblastoma cells and found that PARPis were most effective in increasing the radiation sensitivity of glioblastoma cells in the S-phase (SER50 = 1.60) compared to enriched cell populations in G1 (SER50 = 1.27) or G2 (SER50 = 1.33) phases (Dungey et al., 2008). Tang et al. analyzed the impact of a PARPi, namely, rucaparib, on the proliferation and radiation sensitivity of cervical cancer cells, concluding that the combination of rucaparib and radiotherapy more effectively inhibited cervical cancer cell proliferation and induced G2/M phase arrest than rucaparib alone (Tang et al., 2019). Masutani et al. observed that PARPi 3-aminobenzamide (3-AB) suppressed G1 phase arrest and enhanced G2 phase arrest post-gamma-ray irradiation, potentially by mediating the expression of WAF1/CIP1/p21 and MDM-2 mRNA, thereby participating in the G1 phase signal transduction pathway (Masutani et al., 1995). Meanwhile, Hirai et al. examined the impact of PARPi on the MIA cell line of human pancreatic cancer following exposure to gamma radiation (low LET) and carbon ion radiation (high LET). The findings suggested that PARPis amplified the cellular response to gamma and carbon ion radiations by driving S-phase arrest and locally delaying DBSs through enhanced DNA damage response (DDR) (Hirai et al., 2012).

### 4.3 Tissue level—impact on the tumor microenvironment (TME)

The IR effect on cells is profoundly dependent on the presence of oxygen. Most solid tumors generate hypoxic regions, which is a significantly contributing to tumor radiation resistance. Combining PARPis and radiotherapy ameliorated hypoxia-induced radiation resistance. On the one hand, several PARPis, including olaparib, can induce vasodilation and increase tumor perfusion, effectively enhancing the hypoxic and radiation-resistant components of the tumor, rendering the tumor more sensitive to radiation (Borst et al., 2020). On the other hand, PARPis manifest radiosensitizing effects in hypoxic cells even without any vascular system improvement (Jiang et al., 2016). This could be ascribed to the genetic instability caused by hypoxia through mutational phenotypic effects associated with a decrease in protein expression involved in homology-dependent recombination repair(HRR), which could facilitate “SL” for radiation synergy (Kaplan and Glazer, 2020).

## 5 Radiation therapy reduces resistance to PARPis

Resistance to PARPis is a common occurrence in cancer management. PARPis can block PARP enzyme activity, repressing DNA repair and inducing cancer cell death. However, cancer cells can circumvent PARPis through multiple mechanisms, culminating in resistance. For instance, cancer cells may increase alternative DNA repair pathways to compensate for PARP repair loss, reducing the risk of cell death (Bieńkowski et al., 2022). Moreover, cancer cells may also evade the PARP inhibitory effect by altering PARP gene expression or mutation.

Radiation therapy can diminish resistance to PARPis through several mechanisms. First, radiation therapy can contribute to DNA DSBs, enhancing the sensitivity of cancer cells to PARPis. PARPis can impede SSB repair, leading to the formation of DSBs and elevating the cancer cell sensitivity to radiation therapy. Furthermore, radiation therapy can affect the TME, enhancing PARP inhibitory effects (Monjazeb et al., 2020). Radiotherapy can increase DNA damage within tumor cells, inducing cell death and activating the immune system to increase apoptosis and immune-mediated cytotoxicity (Brandmaier and Formenti, 2020).

Second, radiation therapy can obstruct DNA repair pathways, reducing resistance to PARPis (Deng et al., 2022). PARPis inhibit HR repair, thereby increasing the reliance of cancer cells on other repair pathways. Various types of DNA damage are induced, disrupting HR repair and other repair pathways. This further augments the sensitivity of cancer cells to PARPis (Deng et al., 2022).

Finally, radiation therapy can amplify the effects of PARPis through synergistic effects, reducing resistance occurrence. Radiation therapy and PARPis operate through distinct mechanisms and can enhance tumor cell death and apoptosis at different levels, thereby elevating treatment efficacy. The concurrent administration of PARPis and radiation therapy can limit tumor evasion, thereby decreasing the risk of recurrence and metastasis (Qiu et al., 2022).

Therefore, radiation therapy can mitigate resistance to PARPis and intensify treatment efficacy. This treatment approach also elevates cancer cell sensitivity to PARPis via multiple mechanisms while minimizing the escape mechanisms of tumor cells and lowering the treatment failure rate.

## 6 Clinical research progress of PARPis combined with radiotherapy

The FDA has approved PARPis for the management of ovarian, breast, and prostate cancers with BRCA mutations after the confirmation of distinctive anti-neoplastic effects in numerous Phase III clinical trials. According to preclinical data, PARPis have shown potential as a combination therapy with radiation therapy owing to their ability to enhance the radiation sensitivity of tumor cells, in addition to their use as a monotherapy, Furthermore, earlier clinical trials have explored the combination of PARPis and radiation therapy, suggesting the possibility of superior treatment outcomes for various cancer types.

### 6.1 Preclinical studies

In 2004, Chalmers et al. corroborated that PARPis enhance the radiosensitivity of glioma cells. Moreover, the degree of radiation sensitization by PARPis may rely on the tumor cell cycle distribution during irradiation (Chalmers et al., 2004). In the same year, Calabrese et al. from Newcastle University observed that the combination of radiation and PARPis could significantly decrease the survival rate of colorectal cancer cells. In a nude mouse xenograft model, intraperitoneal injection of PARPi (AG14361) alone did not affect tumor growth compared with the control group injected with saline. However, after injecting the PARPi for 30 min, local X-ray irradiation (2 Gy) was performed. Interestingly, the tumor growth of mice was delayed by 37 days, while the delay from local irradiation alone was only 19 days. The use of combination therapy in mice did not generate significant toxic side effects (Calabrese et al., 2004).

In 2011, experiments on various human head and neck tumor cells, such as UM-SCC1, UM-SCC5, UMSCC6, and FaDu, were conducted by Nowsheen et al., which revealed increased cytotoxicity with the combination of IR and ABT-888 compared to either therapy alone. This increased susceptibility correlated with reduced nuclear EGFR, attenuation of NHEJ, and persistence of DNA damage following IR (Nowsheen et al., 2011).Similarly, a study by Güster et al., in 2014 revealed that inhibiting PARP could enhance the responsiveness of HPV-positive HNSCC cells to radiation therapy, and similar outcomes were detected in laboratory settings. In order to confirm the radiosensitizing effect of the treatment, Khan et al. carried out experiments on female nude mice with HNSCC human xenografts using a novel PARPi termed GPI-15427. The subsequent findings indicated that the utilization of GPI-15427 in combination therapy resulted in a significant decrease in tumor volume compared to radiation therapy alone and the control groups. Furthermore, the cells exhibited heightened DNA damage and raised apoptosis bith *in vitro* and *in vivo*, suggesting that the PARP-1 inhibitor GPI-15427 could enhance the susceptibility of HNSCC tumors to radiation therapy (Güster et al., 2014).

Subsequent studies on the radiosensitizing effect of PARPis have yielded positive outcomes. In 2015, under normoxic and hypoxic conditions, Zhan et al. compared the effects of a PARPi (olaparib) on the radiation sensitivity of esophageal cancer cells. The results indicated that olaparib could significantly increase radiation-induced apoptosis under hypoxic conditions compared to normoxic conditions. The HRR pathway may be blocked under hypoxic conditions, and radiation combined with PARPis can lead to SL (Zhan et al., 2016). Tuli et al. utilized a mouse prostate orthotopic transplantation tumor model to establish that PARPis (Veriparob) in conjunction with radiotherapy can delay tumor growth by 39 days. In contrast, monotherapy and radiotherapy delayed tumor growth by 8 and 30 days, respectively. The 60-day survival rate of mice in the combination group was 40%, while that in the monotherapy and radiotherapy groups was 0. The study also evinced that PARP activity significantly increased following radiotherapy. Moreover, PARP was deactivated after the administration of PARPis, and DNA damage was increased (Tuli et al., 2014).

Mao et al. observed that olaparib improved the radiosensitivity of cholangiocarcinoma (CCA) cells, and this effect was potentiated with incremental olaparib doses. Olaparib enhanced the radiation effect by inhibiting PARP1, inducing DNA damage, and increasing cell apoptosis (Mao et al., 2018).

Shun-Ichiro Kageyama et al. reported the capacity of PARPis to intensify the impact of proton beam therapy (PBT) on esophageal cancer cell lines refractory to radiation and cisplatin. After subjecting OE-21 and KYSE-450 esophageal squamous cell carcinoma cell lines to PARPis for 1 h, the team proceeded to irradiation. In order to delineate the cell survival curve and determine the sensitizing effect ratio (SER), a clonogenic assay was employed to evaluate the impact of multi-fractionated irradiation by comparing 8 Gy/1 fraction and 8 Gy/4 fractions. Additionally, immunofluorescence was utilized to identify foci of γH2AX, Rad51, BRCA1, BRCA2, and 53BP1. The study revealed that PBT in esophageal cancer cells, which were resistant to platinum and radiation, exhibited a significant increase in sensitivity upon treatment with olaparib. The sensitizing effect was observed to range between 1.5-1.7, comparable to the effect of multi-fractionated irradiation. Moreover, the additive effect of olaparib and PBT resulted in an increase in the expression levels of HR- and DSB-related genes. Nonetheless, no additional impact was detected on NHEJ-related genes. Collectively, these results suggested that olaparib is a promising sensitizing agent for PBT in esophageal cancer cells exhibiting resistance (Kageyama et al., 2020).

The impact of olaparib on cellular PARP activity under both aerobic and hypoxic conditions and its ability to enhance the sensitivity of radiation therapy in prostate cancer cells were examined by Gani et al. *In vitro*, PARP activity was inhibited by olaparib under both hypoxic and aerobic conditions. 22Rv1 cells from the prostate cancer cell line were also sensitized to radiation therapy under acute hypoxic, chronic hypoxic, and aerobic conditions by olaparib. In addition, the combination of olaparib and fractionated radiation therapy caused a considerable delay in growth and clonogenic killing *in vivo* without any observed rise in intestinal toxicity. According to the findings, olaparib can improve the effectiveness of radiation treatment in individuals with prostate cancer, irrespective of oxygenation status (Gani et al., 2015).

Certain studies on PARPi radiosensitization have identified that PARPis exert radiosensitizing effects even in normal HRR tumors. Feng et al. revealed that PARPis increased the radiation sensitivity of breast cancer cells, regardless of the BRCA1 mutation status (Feng et al., 2014). At the same time, Bi et al. observed that the PARPi, olaparib, exerted radiosensitizing effects on both BRCA1-normal and BRCA1-mutant high-grade serous ovarian carcinoma (HGSOC) cell lines in an *in vitro* study. However, the radiosensitizing effect was more pronounced on BRCA1-mutant HGSOC cells than on BRCA1-normal cells (Bi et al., 2018). Research on CCA, melanoma, head and neck squamous cell carcinoma, and soft tissue sarcoma has also characterized PARPis as radiosensitizers with potential application value beyond homologous recombination deficiency(HRD) tumors. Their effects involve inhibiting tumor cell proliferation, decreasing the cell clone formation rate, increasing G2/M phase blockade, enhancing DNA DSBs, intensifying the radiation resistance of hypoxic cells, delaying tumor growth, and elevating mouse survival rates (Mangoni et al., 2018; Wang et al., 2020; Weigert et al., 2020; Zhu et al., 2020).

The mechanism by which PARPis exert radiosensitizing effects in normal HRR tumors remains unclear despite having been the subject of extensive research. Laird et al. at the Memorial Sloan Kettering Cancer Center in the United States utilized two distinct PARPis and discovered that veliparib did not possess radiosensitizing properties at the same concentration with the same enzyme inhibitory effect, whereas talazoparib showed a significant radiosensitizing effect. Thus, inhibitors with a stronger PARP trapping ability could induce more DNA DSBs, ultimately improving tumor cell radiation sensitivity (Laird et al., 2018).

### 6.2 Clinical research

#### 6.2.1 Head and neck tumors

Karam et al. conducted a phase I trial in which olaparib, cetuximab, and radiotherapy were administered to heavy smokers with locally advanced head and neck cancer. Olaparib orally administered twice daily, the Maximal tolerance dose(MTD) was 50 mg. However, the recommended phase II dose was 25 mg, administered orally twice daily. Noteworthily, the most common treatment-related grade 3–4 side effects were radiation dermatitis and mucositis (38% and 69%, respectively). The median survival rate was 37 months, and the 2-year survival rates were 72% for overall survival, 63% for progression-free survival, 72% for local control, and 79% for distant control. Recurrence rates were higher in patients who continued smoking. Lastly, gene analysis revealed potential correlations with MYC and KMT2A (Karam et al., 2018).

#### 6.2.2 Breast cancer

Feng et al. (2014) observed that PARP1 inhibitors induced varying levels of radiosensitization across different breast cancer cell lines, irrespective of the breast cancer subtype or the presence of BRCA1 gene mutation. Of note, PAR formation levels could be used to predict the level of radiosensitization within 24 h of initiating treatment. Thus, PAR has the potential to serve as a biological marker for early treatment response and identify individuals who could benefit from a combination of PARP1 inhibitors and radiation therapy (Feng et al., 2014).

Jagsi et al. performed a phase I study (TBCRC 024) combining veliparib with chest wall and lymph node radiation in patients with inflammatory or locally recurrent breast cancer. Five dose-limiting toxicities (DLTs) were identified in the study, with a crude rate of 10% for any grade 3 toxicity at year 1, 16.7% at year 2, and 46.7% at year 3. Severe fibrosis developed in 6 of the 15 surviving patients at year 3. Long-term toxicity monitoring is essential in radiosensitizer trials, considering that nearly half of surviving patients experienced grade 3 adverse events at 3 years (Jagsi et al., 2018).

The safety and tolerability of the combination of olaparib and radiotherapy in triple-negative breast cancer (TNBC) patients were evaluated in a study by Loap et al. The maximum dose of 200 mg administered twice daily was well-tolerated without any DLT. Although long-term results are pending, this dose of olaparib is recommended for future trials. After a 1-year follow-up, the toxicity results showed that the combination of olaparib and radiotherapy was well-tolerated in TNBC patients, with no adverse events exceeding grade 3; skin toxicity was minimal (Loap et al., 2021). At the recent 2022 American Society of Clinical Oncology (ASCO) annual meeting, the final results of the RADIOPARP phase 1 trial, which evaluated the safety and tolerability of the combination of olaparib and breast radiation therapy in TNBC patients, were presented. 24 patients were enrolled in the study between 2017 and 2019, with olaparib escalated to 200 mg twice daily without DLT. With a median follow-up of 34 months, no late treatment-related toxicities of grade 3 or higher were recorded. The most severe toxicities recorded were grade 2 breast pain, fibrosis, deformity, and telangiectasia. Olaparib was well-tolerated as a radiosensitizer in TNBC patients, and an olaparib dose of 200 mg twice daily could be considered in future trials evaluating this combination (Loap et al., 2022).

#### 6.2.3 Non-small cell lung cancer(NSCLC)

A phase I trial to assess olaparib in combination with high-dose radiotherapy, with or without concurrent cisplatin, in NSCLC patients was conducted. Olaparib was administered to 28 patients, either once or twice daily, at a dose of 25 mg. In case of hematologic and late esophageal toxicities, an MTD of 25 mg once daily without the use of cisplatin would be applicable. An extended follow-up of the 20 patients who were still alive at the 1-year mark revealed severe late pulmonary adverse events in 5 patients at a latency of 1–2.8 years, including bronchial strictures, pulmonary fibrosis, and fatal hemorrhage. A notable observation was the high incidence of pulmonary fibrosis in patients treated with PARPis. At the MTD, a decrease in PAR levels exceeding 95% and the eradication of PARylation caused by radiation was observed in patients administered olaparib. Moreover, a median overall survival (OS) of 28 months and a 2-year local control rate of 84% were observed in patients with locally advanced NSCLC after a median follow-up of 4.1 years. Excessive toxicity was associated with the combination of daily low-dose cisplatin, olaparib, and concurrent mild dose-split radiotherapy. Therefore, the exploration of alternative, more conformal radiotherapy schedules is imperative to enhance lung and esophagus protection (de Haan et al., 2021).

The combination of veliparib and chemoradiotherapy was analyzed in a randomized placebo-controlled phase II trial enrolling stage III NSCLC patients. DLT was observed in two of the 21 patients who received 40 mg and 80 mg of veliparib twice daily in the phase I study. One participant in the 40 mg cohort experienced severe esophagitis with dysphagia, while another participant in the 80 mg cohort developed severe esophagitis with dehydration. In the experimental group, 31 patients received 120 mg of veliparib twice daily, and the response rates were 56% and 69% in the veliparib and placebo groups, respectively. However, there were no significant differences in progression-free survival (PFS) between the veliparib and placebo groups; for the veliparib and placebo arms, the 1-year PFS rates were 47% and 46%, and the 1-year OS rates were 89% and 54%, respectively. Despite the early termination of the study, the findings inferred that the combination of CRT and veliparib was feasible and well-tolerated, although the precise efficacy could not be determined. The evaluation of PARPis in lung cancer is considered promising, especially with advancements in predictive biomarkers and combination immunotherapy (Argiris et al., 2021).

#### 6.2.4 Pancreatic cancer

In a phase I clinical trial conducted by Tuli et al., the combination of veliparib, gemcitabine, and radiotherapy was investigated for the treatment of locally advanced pancreatic cancer. The MTD of veliparib was identified as 40 mg BID, with 16 DLTs observed among 12 patients. Grade 3 or higher adverse events included lymphopenia (96%) and anemia (36%). The median OS was 15 months; however, cases with DDR pathway gene alterations exhibited a longer survival of 19 months, while a shorter survival of 14 months was recorded for those with intact cases. Although no significant associations were observed between outcomes and PAR, TMB, or MSI levels, the transcriptomes of the PARP3 and RBX1 in the DDR pathway were significantly related to OS (Tuli et al., 2019).

#### 6.2.5 Glioblastoma

Mueller et al. discovered that the combination of niraparib and radiation treatment decreased clonogenicity and had an additional effect on radiation in neuroblastoma cell lines. The administration of this combination therapy to high-risk neuroblastoma patients resulted in higher survival rates compared to single-treatment modalities (*p* < 0.01). The effectiveness of this approach was further bolstered by the rise in cleaved caspase-3 and γ-H2AX levels observed in the tumors of patients in the combination therapy group. These results implied that the combination of niraparib and radiation therapies could be a promising approach for the treatment of high-risk neuroblastoma in the pediatric population (Mueller et al., 2013).

Hao Wen Sim et al. reported the outcomes of a multicenter phase II trial (VERTU) that assessed the use of veliparib, radiotherapy, and temozolomide for the treatment of newly diagnosed glioblastoma patients with unmethylated MGMT promoter status. In the experimental group, veliparib, radiotherapy, and adjuvant veliparib and temozolomide were administered, whereas in the control group, concurrent temozolomide and radiotherapy were given after adjuvant temozolomide. The primary endpoint was to prolong the 6-month progression-free survival (PFS-6m). The experimental arm had a PFS-6m of 46%, while the standard arm had a PFS-6m of 31%, with a total of 125 participants enrolled. Thrombocytopenia and neutropenia were the most frequent grades 3-4 adverse events. Although the regimen containing veliparib was feasible and well-tolerated, there was insufficient evidence to demonstrate clinical benefits in this population (Sim et al., 2021).

#### 6.2.6 Rectal cancer

In a phase Ib clinical trial for locally advanced rectal cancer, the safety and tolerability of the combination of veliparib, capecitabine, and radiation therapy were assessed. A total of 32 patients received veliparib, 22 in the dose escalation group and 10 in the safety expansion group. There were no observed grades 3-4toxicities, and the recommended dose for phase II was established as 400 mg taken twice daily. Nausea, diarrhea, and fatigue were the most frequent adverse effects associated with the treatment regimen. Of the 31 patients evaluated, 22 experienced a reduction in tumor size post-surgery, while nine attained a full pathological response. The pharmacokinetics of veliparib were dose-dependent and had no impact on that of capecitabine. Grade 4 adverse events were not observed; however, three patients experienced grade 3 diarrhea (Czito et al., 2017).

#### 6.2.7 Brain metastases

Veliparib, a PARPi that crosses the blood-brain barrier, enhances the anti-tumorigenic activity of radiation therapy. A phase 1 dose-escalation study evaluated the safety and efficacy of combining veliparib with (whole-brain radiation therapy) WBRT in patients with brain metastases. Eighty-one patients underwent treatment with escalating doses of veliparib (10–300 mg, administered twice daily) and WBRT (30.0 or 37.5 Gy in 10 or 15 fractions). The most common adverse events associated with veliparib were fatigue, nausea, and reduced appetite. Preliminary efficacy results indicated better-than-anticipated survival rates, with a median survival time (MST, 95% CI) of 10.0 months for the NSCLC subgroup and 7.7 months for the breast cancer subgroup. Although the study was non-controlled, these results could serve as a foundation for future trials. This was contrasted with the MST predicted by nomogram models of 3.5 months (3.3-3.8) and 4.9 months (4.2-5.5), respectively. No new toxicities were identified following the addition of veliparib to WBRT compared to WBRT alone (Mehta et al., 2015).

#### 6.2.8 Peritoneal carcinomatosis

Resis et al. conducted a phase I clinical trial involving patients with advanced ovarian and fallopian tube peritoneal metastasis cancer to evaluate the effects of combining veliparib with low-dose fractionated whole-abdominal radiation (LDFWAR). The treatment was administered to 32 patients, and the highest tolerated dose was 250 mg, taken orally twice a day. Lymphopenia, anemia, thrombocytopenia, neutropenia, leukopenia, nausea, diarrhea, anorexia, vomiting, and fatigue were the most frequent grade 3 and 4 toxicities. The median PFS was 3.6 months, whilst the median OS was 9.1 months. As anticipated, patients responsive to platinum treatment had a longer OS compared to those not responsive to platinum treatment. All groups experienced a decline in quality of life throughout the treatment course. Of the 18 ovarian cancer patients with confirmed BRCA status, BRCA mutations were present in 5 patients. Among the platinum-sensitive patients with a germline BRCA mutation, only one patient achieved an objective response. Gastrointestinal symptoms, fatigue, and myelosuppression were the most frequent side effects linked with the use of veliparib and LDFWAR; nonetheless, the combination was well-tolerated (Reiss et al., 2017).

#### 6.2.9 Ongoing clinical trials

A Phase I/IIa Study of Concomitant Radiotherapy With Olaparib and Temozolomide in Unresectable High Grade Gliomas (HGG) Patients is currently in the recruitment phase (ClinicalTrials.gov Identifier: NCT03212742), this study investigate the toxicity and efficacy of olaparib and TMZ concomitantly with radiotherapy in first line treatment of unresectable high risk HGG. A study investigates the combination of Dostarlimab and Niraparib plus Radiation Therapy (RT) is safe and effective in participants with metastatic triple negative breast cancer (ClinicalTrials.gov Identifier: NCT04837209), The investigators believe that dostarlimab may inhibit the PD-1 protein on triple negative breast cancer cells, thus allowing the immune cells to recognize and destroy cancer cells. Radiotherapy is a standard-of-care treatment that is given to stop the growth of tumors. Radiotherapy can also stimulate the immune system, which leads to the destruction of tumor cells in the treated areas. Combining radiotherapy with anti-cancer drugs like dostarlimab and niraparib may increase the ability of the immune system to control or destroy cancer cells throughout the body. Another angoing clinical trial will evaluate whether the immune-sensitizing effects of immunotherapy (Pembrolizumab) and radiation with or without a PARP-inhibitor (Olaparib) will increase the effects of immunotherapy in men with high-risk localized prostate cancer http://ClinicalTrials.gov" \o "http://ClinicalTrials.gov"ClinicalTrials.gov Identifier: NCT05568550). The investigators think that Immunotherapy and PARP-inhibitor are known to have radio-sensitizing effects when combined with radiation therapy. In addition, the combination with PARP-inhibitor and radiation can increase neoantigen expression, cytotoxic lymphocyte infiltration within the tumor microenvironment and increased immune stimulating cytokine concentration. Thus, there is a potential synergy of combining immunotherapy and PARP-inhibitor. There are also some ongoing trials, and their trial results will also provide new evidence for the combined application of PARPi and radiotherapy in the future.

All clinical trials of PARPis combined with radiotherapy are summarized in Table 1.

**TABLE 1:** Clinical trials of PARP inhibitors in combination with radiotherapy.

Clinical trial	PARP inhibitor	Clinical status	Localization	Patient number	Radiotherapy plan	ORR	PFS	OS	AEs
Karam et al. (2018)	Olaparib	Phase I Trial	Head and neck	16	69.3 Gy in 33 fractions	local control 72%, distant control 79%	2-year PFS rate 63%	2-year OS rate 72%	dermatitis 38%, mucositis 69%
Jagsi et al. (2018)	Veliparib	Phase I trial	Breast cancer	30	50 Gy + 10 Gy (boost)/25 fr	NA	3-year PFS rate 50%	3-year OS rate 34%	Fibrosis
Loap et al. (2021)	Olaparib	Phase 1 Trial	TNBC	24	50 Gy/25 fr	NA	NA	NA	No grade ≥3 toxicity; few grade 2 skin toxicity
Loap et al. (2022)	Olaparib	Phase 1 Trial	TNBC	24	50.4 Gy/28 fr	NA	3 years event free survival(EFS) rate 65%	3 years OS rate 83%	grade 2 breast pain, fibrosis, and deformity
de Haan et al. (2021)	Olaparib	Phase 1 Trial	NSCLC	28	66 Gy/24 fr	2 years loco control rate 84%	NA	Median OS was 28 months	grade 5 pulmonary AEs was 18%
Argiris et al. (2021)	Veliparib	Phase 2 Trial	NSCLC	21	60 Gy/30 fr	ORR veliparib vs. placebo:56% vs. 69%	1 year PFS veliparib vs. placebo:47% vs. 46%	1 year PFS veliparib vs. placebo:89% vs. 54%	pneumonitis, esophagitis, neutropenia
Tuli et al. (2019)	Veliparib	Phase 1 Trial	Pancreas	30	36 Gy/15 fr	NA	NA	Median OS was 15 months	lymphopenia (96%) and anemia (36%)
Sim et al. (2021)	Veliparib	Phase 2 Trial	Glioblastoma	125	60 Gy/30 fr	NA	6 months PFS rate, experimental vs. standard arms:46% vs. 31%	Median OS, experimental vs. standard arms:12.7 months vs. 12.8 months	Thrombocytopenia, neutropenia
Czito et al. (2017)	Veliparib	Phase 1b Trial	Rectum	32	50.4 Gy/28 fr	NA	NA	NA	nausea, diarrhea, and fatigue
Mehta et al. (2015)	Veliparib	Phase 1 Trial	Brain metastases	81	30 Gy/10 fr	NA	NA	NSCLC was 10.0 months; breast was 7.7 months	Nausea, fatigue, alopecia, headache
Reiss et al. (2017)	Veliparib	Phase 1 Trial	Peritoneal carcinomatosis	32	21.6 Gy/36 fr	NA	median PFS was 3.6 months	median OS was 9.1 months	lymphopenia, anemia, thrombocytopenia, neutropenia, leukopenia, nausea, diarrhea, anorexia, vomiting, fatigue

PARP, poly ADP-ribose polymerase; OS, overall survival; PFS, progression-free survival; TBNC, triple-negative breast cancer; DLT, dose limiting toxicity; EFS, event free survival; NSCLS, non-small cell lung carcinoma; ORR, objective response rate; DDR, DNA, damage response.

## 7 Toxicity for PARP inhibitors combined with radiotherapy

We analized and resume the toxicity of combination of PARPi and radiotherapy. According to the tumor locations, in the head and neck cancer, the most common treatment-related grade 3–4 side effects were dermatitis (38%), mucositis(69%).in Breast cancer, lung cancer, the most frequent AEs were Fibrosis, pneumonitis, esophagitis and skin toxicities. In Pancreas cancer, glioblastoma and peritoneal carcinomatosis presented more hematological toxicities, including lymphopenia, thrombocytopenia, neutropenia and anemia in rectum cancer and brain metastases the most common AEs were nausea, diarrhea and fatigue.

Through the above datas, we found that the AEs of PARPi combined with radiotherapy were feasible and usually safe, but we should ensure that patients suitable for the combined therapy are correctly selected. Optimizing patient selection, RT technique/dose/fractionation and PARP-I dose/timing to attenuate normal tissue responses and improve antitumor efficacy remaind a major challenge.

## 8 Future perspective of combination therapy

The aforementioned article described the results of research involving PARPis and radiotherapy, which indicate that their prospects with immunotherapy are broad.

**FIGURE 3:**
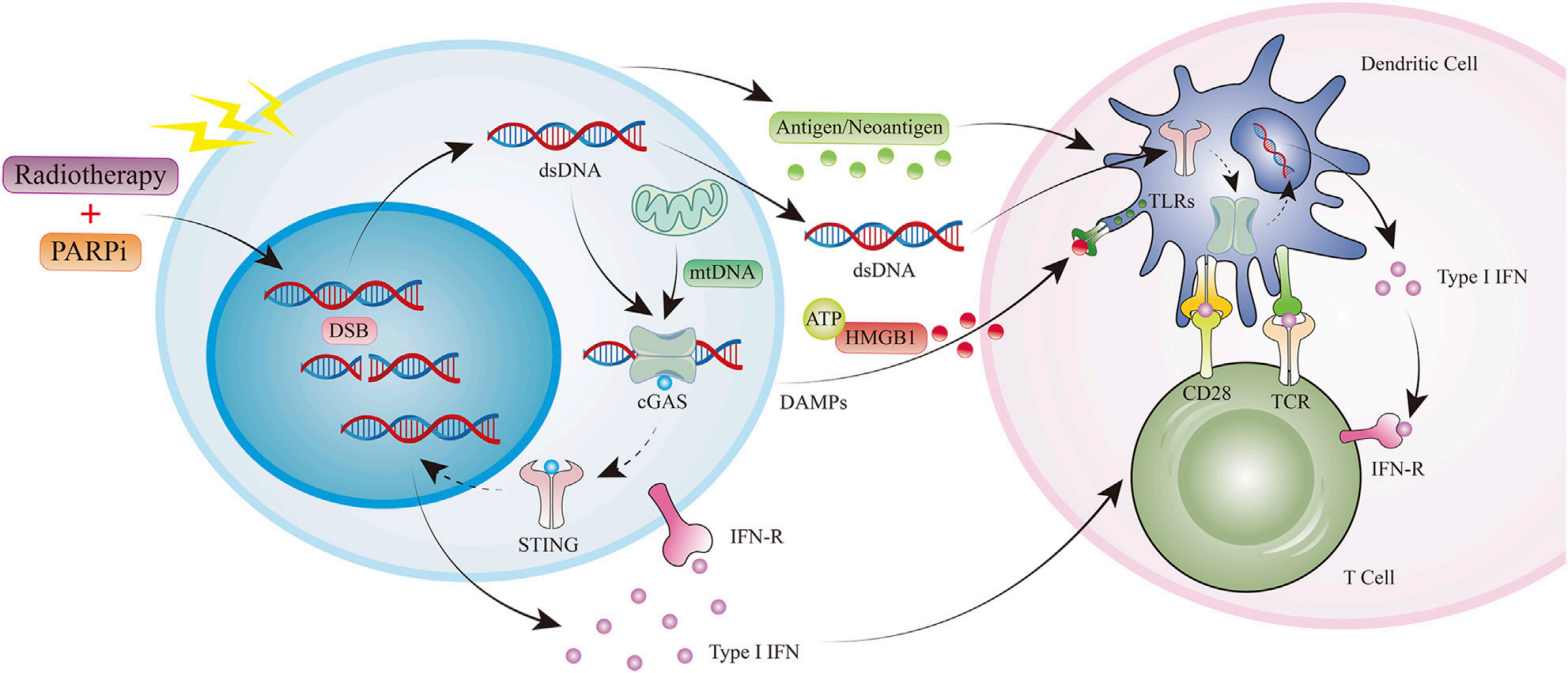
Radiotherapy and PARPi induce antitumor immunity. PARPi, PARP inhibitor; ds DNA, double-stranded DNA; mt DNA, mitochondrial DNA; DAMPs, Damage-associated molecular patterns; HMGB1, High Mobility Group Protein 1; TLR,Toll-like receptor.

Cytokine production, cell recruitment, and mutational burden are influenced by PARPis and radiotherapy, with their synergistic effects more likely in tumors with a deficient DNA damage response. Impaired DNA repair due to mutation or PARPi treatment can further prolong damaged DNA-induced cGAS/STING pathway activation (Figure 3). (Chabanon et al., 2019) In breast cancer, PARPis have been shown to induce interferon (IFN)-I and CCL5 expression in tumor cells via the cGAS-STING pathway (Pantelidou et al., 2019). As with radiation therapy, activation of this pathway by PARPis leads to the recruitment of CD8 T cells at the tumor site. In a BRCA1-deficient ovarian cancer model, IFN-γ and tumor necrosis factor (TNF)-α production by CD8 T cells and natural killer (NK) cells increased following PARPi treatment (Huang et al., 2015). In the same model, inhibition of PARP also induced a decrease in the frequency of MDSCs, which negatively regulated anti-tumor immune responses (Huang et al., 2015). Notably, PARPis protected CD8 T cells from oxygen-free radical-induced apoptosis by inhibiting the nuclear accumulation of apoptosis-inducing factors. Moreover, other immune-related functions were promoted by the sustained release of IFN-I from PARPi in the TME, given that IFN-I activates dendritic cells, maintains the cross-presentation of tumor-derived antigens to T cells, is essential for NK cell-mediated anti-tumor immunity, and activates M1 anti-tumor macrophages in conjunction with TLR4 ligands (such as HMGB1) (Karimi et al., 2015).

Both PARPis and radiotherapy impact cytokine production, cell recruitment, and mutational burden, with synergistic effects more likely in tumors deficient in the DNA damage response. In an EGFR-mutant NSCLC mouse model, niraparib promoted IFN-I production through synergistic effects on the cGAS/STING pathway, thereby increasing radiotherapy-driven anti-tumor immunity. The observed reduction in tumor growth and prolonged survival was associated with increased CD8 T cell infiltration. Furthermore, the expression of major histocompatibility complex (MHC)-I molecules and inflammatory cytokines was increased, and the translocation of calreticulin to the cell surface was promoted in colorectal cancer cells by the combination of veliparib and IR. Interestingly, the combination of PARPi and RT was more effective in microsatellite unstable tumor models (Seyedin et al., 2020).

The 2021 American Association for Cancer Research (AACR) conference reported a preclinical study on the combination of the PAPRi niraparib, radiotherapy, and immunotherapy for small-cell lung cancer (Zhang et al., 2021). A study to explore the capability of niraparib, to act as a radiosensitizer and improve the efficiency of radiotherapy in the management of patients diagnosed with EGFR-mutant NSCLC was undertaken by N. Zhang et al. In order to assess the impact of niraparib and radiation on tumor cells, the team employed techniques such as clonogenic and apoptotic assays, immunofluorescence staining, real-time fluorescence quantitative polymerase chain reaction, and immunoblotting. Furthermore, a mouse model with a functional immune system was constructed to validate their discoveries *in vivo*. The study demonstrated, both *in vitro* and *in vivo*, that the STING/TBK1/IRF3 pathway was activated by the combination of radiation and niraparib, which effectively suppressed tumor growth and increased the levels of INF-β, CCL5, and CXCL10, as well as the proportion of CD8^+^ T lymphocytes. Collectively, the findings suggest that PARPis could improve the immune response against tumors and serve as a radiosensitizer for EGFR-mutant NSCLC (Zhang et al., 2021).

## 9 Conclusion and limitations

Therefore, PARP inhibitors have successfully treated BRCA-mutated and platinum-sensitive recurrent ovarian cancer. In contrast, the combination of PARP inhibitors and radiation therapy is still nascent. Although preliminary clinical trial data is encouraging, many issues need further research. (1) The safety and efficacy of long-term drugs remain unclear. Alotaibi et al. from Virginia Tech observed that combination therapy only enhanced tumor growth arrest rather than promoting tumor cell death. Therefore, further observation is required to determine whether this combination therapy improves long-term survival (Alotaibi et al., 2016). Additionally, the effect of PARP inhibitors on highly proliferative non-tumor tissues, including mucosa or bone marrow, remains unclear. Some studies have observed an increased risk of abnormal bone marrow proliferation or acute myeloid leukemia when utilizing PARP inhibitors (Alotaibi et al., 2016). (2) More clinical trials should investigate the optimal dose, timing, and adverse reaction prevention of PARP inhibitors associated with radiation therapy. (3) In clinical practice, using PARP inhibitors and radiation therapy simultaneously or radiation therapy after PARP inhibitor treatment is challenging and unclear. (4) It is urgent to find reliable biomarkers to screen for patients who can benefit from this combination therapy and predict patient efficacy while implementing individualized precision treatment. For instance, among the many genes associated with the homologous recombination repair pathway, can the gene status such as ATM, ATR, PALB, and FANC, other than BRCA1/2 mutations, predict the efficacy of PARP inhibitors? Therefore, with the continuous advancement of research, this combination therapy can deliver more extensive therapeutic effects.
